# Evaluation of quantitative synchrotron radiation micro-X-ray fluorescence in rice grain

**DOI:** 10.1107/S1600577523000747

**Published:** 2023-02-15

**Authors:** Matt A. Limmer, Samuel M. Webb, Angelia L. Seyfferth

**Affiliations:** a Department of Plant and Soil Sciences, University of Delaware, Newark, DE 19716, USA; b Stanford Synchrotron Radiation Lightsource, SLAC National Accelerator Laboratory, Menlo Park, CA 94025, USA; ESRF – The European Synchrotron, France

**Keywords:** arsenic, metal(loid)s, rice bran, rice grain, synchrotron radiation microprobe X-ray fluorescence, SR-µXRF

## Abstract

Concentrations of nutrients and contaminants in rice grain affect human health, specifically through the localization and chemical form of elements. Methods to spatially quantify the concentration and speciation of elements are needed to protect human health and characterize elemental homeostasis in plants. Here, an evaluation was carried out using quantitative synchrotron radiation microprobe X-ray fluorescence (SR-µXRF) imaging by comparing average rice grain concentrations of As, Cu, K, Mn, P, S and Zn measured with rice grain concentrations from acid digestion and ICP-MS analysis for 50 grain samples. Better agreement was found between the two methods for high-*Z* elements. Regression fits between the two methods allowed quantitative concentration maps of the measured elements. These maps revealed that most elements were concentrated in the bran, although S and Zn permeated into the endosperm. Arsenic was highest in the ovular vascular trace (OVT), with concentrations approaching 100 mg kg^−1^ in the OVT of a grain from a rice plant grown in As-contaminated soil. Quantitative SR-µXRF is a useful approach for comparison across multiple studies but requires careful consideration of sample preparation and beamline characteristics.

## Abstract 2

This work demonstrates which elements are the most easily quantifiable by synchrotron radiation for microprobe X-ray fluorescence (SR-µXRF) imaging in order to understand elemental distributions in plant tissues, with a focus on metals and metalloids in rice grain. This work provides SR-µXRF users with data to understand which elements can be reliably quantified and guidance on how to consider the limitations of the technique to most effectively interpret such data.

## 1. Introduction

The concentration, speciation and localization of elements in plant tissues have implications for both plant survival and consumers of plants. Understanding the localization of toxic and nutrient elements can provide mechanistic insight into plant tolerance and homeostasis (Conn & Gilliham, 2010 ▸). Therefore, measurements of elemental species and/or localization must also be quantitative. To quantify concentrations of elements, plant parts are typically homogenized, digested and the liquid digest quantitatively analyzed. Obtaining a spatially finer understanding of elemental concentrations via digestion is thus limited to the resolution of dissection, which becomes time-consuming or impractical at smaller scales. Mechanistic understanding requires measurement of the concentrations and/or flows of elements at the scale of the governing phenomena, which may range from tissue to sub-cellular (*i.e.* millimetre to nanometre). Thus, methods to quantitatively measure the concentration distribution are needed to advance our mechanistic understanding of elemental homeostasis and cycling.

Several techniques exist for determining the localization of elements and nutrients in plants; each has advantages and disadvantages. Laser-ablation coupled with inductively coupled mass spectrometry (LA-ICP-MS) allows for quantitative elemental mapping in plant tissues at 10–100 µm resolution, depending on the spot size and sensitivity that the system can achieve. However, quantification can suffer from matrix effects requiring matrix-matched standards, isotope dilution or calibration with doped gels (Becker *et al.*, 2008 ▸; Pozebon *et al.*, 2017 ▸; Bauer *et al.*, 2019 ▸; Pan *et al.*, 2022 ▸). High-resolution secondary ion mass spectrometry (SIMS, *e.g.* nanoSIMS) can quantify a wide variety of elements at low concentrations and at sub-cellular spatial resolution (50 nm), but accuracy is affected by the matrix, and few nanoSIMS instruments currently exist (Moore *et al.*, 2012 ▸). NanoSIMS also has the advantage of resolving isotopes and mapping light elements such as carbon (de Samber *et al.*, 2020 ▸). Synchrotron radiation microprobe X-ray fluorescence (SR-µXRF) imaging is a widely used, non-destructive technique to measure elemental localization in plant tissues at the micrometre (SR-µXRF) to nanometre (SR-nanoXRF) scale (Kopittke *et al.*, 2014 ▸; Punshon *et al.*, 2009 ▸; Seyfferth *et al.*, 2011 ▸; Zhao *et al.*, 2014 ▸). Elements investigated in plants by SR-µXRF include As, Ca, Cd, Co, Cr, Cu, Fe, K, Mg, Mn, Ni, P, S, Se, Si, Tl and Zn, typically in the mg kg^−1^ range (Punshon *et al.*, 2009 ▸). A major advantage of SR-µXRF over LA-ICP-MS or nanoSIMS is that it can be combined with microprobe X-ray absorption spectroscopy (µXAS) or X-ray absorbance near-edge structure (XANES) imaging to provide elemental speciation, which is particularly important for elements that exist in various forms. However, for heavier elements, SR-µXRF may be less sensitive and accurate than LA-ICP-MS, especially when the incident X-ray beam cannot reach the *K*-edge of the desired element (*e.g.* tungsten and cadmium), requiring the use of the *L*- or *M*-edge that yield fewer fluorescent X-rays and may overlap with fluorescent X-rays from the *K*-edge of lighter elements (VanderSchee *et al.*, 2020 ▸). Additionally, despite SR-µXRF being a powerful tool for answering questions of elemental localization and speciation, it is generally considered qualitative or semi-quantitative. To date, there have been few attempts to evaluate the quantitative robustness of this technique.

Quantitative SR-µXRF is seldom attempted, but quantitative bulk XRF with benchtop units has been reported. Portable or benchtop energy-dispersive XRF (ED-XRF) instruments have been used to quantify Zn, Fe, Ca and Cd in rice grain, and good correlations have been found between ED-XRF and ICP-OES (Paltridge *et al.*, 2012 ▸; Guild & Stangoulis, 2022 ▸; Taleon *et al.*, 2020 ▸; Li *et al.*, 2018 ▸; Yada *et al.*, 2006 ▸), suggesting that quantitative SR-µXRF is possible for rice grain. Quantifying elements in any type of sample by SR-µXRF generally requires either matrix- and thickness-matched standards or, more commonly, standards in a different matrix, such as a thin film. Using standard thin films yields areal concentrations, which are simpler but less informative and less comparable with bulk measurements. Additionally, areal concentrations are problematic for non-flat samples, samples thick enough to attenuate the emitted fluorescence for the element of interest and samples of variable composition, resulting in semi-quantitative measurements. Although not reported in plants, fully quantitative SR-µXRF has been successful in other matrices. In an early method development paper, Mavrogenes *et al.* (1995 ▸) quantified Sr content in fluid inclusions in quartz, determining a method detection limit of ∼2000 p.p.m. Wang *et al.* (2010 ▸) quantified Ca, Cu, Fe and Zn in a mouse brain using certified reference materials and normalizing by Compton scattering. Iron concentrations in human brains measured by SR-µXRF were comparable with ICP-MS measurements (Zheng, Nichol *et al.*, 2013 ▸) by quantifying with Fe thin films and correcting for fluorescence attenuation with sample thickness (Hopp *et al.*, 2010 ▸).

Numerous researchers have used SR-µXRF to investigate the localization of As species in rice (*Oryza sativa L.*) because of concerns surrounding human exposure via consumption. Unlike hyperaccumulators, where As levels are sufficiently high to overcome detection limitations, levels of As in rice grain are low (∼0.2 mg kg^−1^). However, the various As species present in the environment and the numerous As–plant interactions make SR-µXRF an ideal tool for this system. Beginning in the soil, SR-µXRF has been used to demonstrate As and Fe co-localization in Fe-rich root plaques (Kramar *et al.*, 2017 ▸; Neumann *et al.*, 2017 ▸; Seyfferth *et al.*, 2010 ▸, 2011 ▸; Smith *et al.*, 2009 ▸; Yamaguchi *et al.*, 2014 ▸; Limmer *et al.*, 2021 ▸). SR-µXRF has also been used to study interactions of As with rice roots. Kopittke *et al.* (2014 ▸) collected XANES spectra at each SR-µXRF pixel of hydro­ponic rice roots and found that arsenate was reduced to As(III)-thiol complexes as the As(V) moved into the root. Seyfferth *et al.* (2017 ▸, 2021 ▸) reported As accumulation in lateral root junctions and used SR-µXRF at multiple incident beam energies to parameterize maps of arsenite and arsenate. In above-ground tissues, SR-µXRF has been used to investigate the elemental distribution in rice leaves (Wu *et al.*, 2016 ▸; Zheng *et al.*, 2011 ▸), internodes (Moore *et al.*, 2014 ▸; Smith *et al.*, 2009 ▸; Yamaoka *et al.*, 2010 ▸; Zheng *et al.*, 2011 ▸) and nodes (Chen *et al.*, 2015 ▸; Moore *et al.*, 2014 ▸; Yamaguchi *et al.*, 2012 ▸; Zheng *et al.*, 2011 ▸). Of most importance for human health, SR-µXRF has been used to examine As and other elements in rice grain. Carey *et al.* (2011 ▸, 2012 ▸) performed SR-µXRF microtomography on rice grains exposed to different forms of As and Se. Lombi *et al.* (2009 ▸) and Kyriacou *et al.* (2014 ▸) analyzed 70 µm-thick grains for As, Cu, Fe, Mn and/or Zn. Lu *et al.* (2013 ▸) analyzed 200 µm-thick rice grains and germinating rice grains to identify elemental remobilization during germination. Meharg *et al.* (2008 ▸) and Muehe *et al.* (2019 ▸) imaged grains split in half, while Zheng *et al.* (2011 ▸) imaged whole grains during development to identify As localization. Zheng *et al.* (2013 ▸) measured As localization in hydro­ponically grown plants using hand sections of grains divided longitudinally in half or cross-sections from the middle third of the grain. Though these reports provide information about elemental localization at varying levels of detail, quantification is relative, and differences in sample preparation and beamline characteristics can hinder comparisons between studies.

This study aimed to determine the accuracy of SR-µXRF in quantifying the concentration of elements in rice grain. To test this, thin sections were quantified and averaged by SR-µXRF and compared with bulk concentrations measured by ICP-MS after acid digestion. Fluorescence signals were integrated by two different methods to examine their effect on quantification. We also determined the reproducibility of the SR-µXRF method and generated quantitative SR-µXRF maps of elements in rice grains. Method detection limits were estimated, and application considerations were discussed. We report that SR-µXRF can be performed quantitatively in rice grain for Zn, Cu, As and Mn.

## 2. Materials and methods

### 2.1. Rice grain

Unpolished rice grain (*i.e.* brown rice) was obtained from 33 unique treatment combinations from several different hydro­ponic, pot and field studies. Hydro­ponic studies included experiments with and without arsenic added to the media (Limmer, Wise *et al.*, 2018 ▸; Limmer & Seyfferth, 2020 ▸), while other media constituents were generally held constant. Growth chamber pot studies included soils either moderately contaminated with As [16 mg kg^−1^ soil As (Seyfferth *et al.*, 2016 ▸)] or spiked with arsenic [25 mg kg^−1^ As (Teasley *et al.*, 2017 ▸)] and amended with different Si-rich materials. A field study with Si-rich amendments with soil As at background levels (5 mg kg^−1^ As) in Delaware was also included (Limmer, Mann *et al.*, 2018 ▸; Limmer & Seyfferth, 2021 ▸). Rice varieties included three long grains – ‘IR66’, ‘Jefferson’ and ‘Lemont’ – and one medium grain – ‘M206’. All grains were mature and air-dried.

#### 2.1.1. Rice grain ICP-MS analysis

Bulk elemental analysis of the dehusked, unpolished rice grain followed previously published methodology (Seyfferth *et al.*, 2016 ▸). For each treatment combination, ∼400 mg of finely ground rice grain was digested in 7 ml of trace metal grade concentrated nitric acid via microwave digestion (Mars 6 Express, CEM Corporation). The vessels were ramped to 200°C over 20 min and held for 10 min. The digested solutions were diluted to 4% acid and analyzed with ICP-MS (Agilent 7500cx) operating in He collision mode. Certified rice flour (NIST 1568a) and acid blanks were included with the digestion. Recovery of the reference material was acceptable for As (103–110%), Cu (79%), Fe (75–84%), K (88–91%), Mg (77–80%), Mn (92%), P (86–87%), S (88–92%) and Zn (88%) (*n* = 2).

#### 2.1.2. Rice grain thin sections

For SR-µXRF measurements, 30 µm-thick grain sections were prepared. Individual grains (4–6) were first embedded in EPO-TEK 301-2FL ep­oxy, taking care to orient the grain for ease of sectioning. Typically, three grains were placed vertically (for cross-sections) into a small piece of foam, while two grains were laid on their side (for longitudinal sections). For samples from pot studies (soil or hydro­ponic), each grain on the slide came from the same plant, while grains on the same slide from the field site were from the same treatment combination. Thin sections were prepared by Spectrum Petrographics Inc. (Vancouver, WA, USA) under low-oxygen conditions. Rice grains were confined to a monolayer on a quartz slide and processed using universally standard thin sectioning methods, including surface preparation, mounting to quartz, cutoff, grinding, lapping and polishing. Section types included cross-sections through the center of the grain and longitudinal sections through the center of the grain.

#### 2.1.3. Rice grain SR-µXRF measurements

SR-µXRF measurements were conducted on 50 rice grains at Stanford Synchrotron Radiation Lightsource (SSRL) using beamline 10-2 in two separate experiments. The beamline was equipped with a 30 pole, 1.45 Tesla wiggler insertion device and a double-crystal Si(111) monochromator. A pinhole aperture created a spot size of 25 µm. The sample was rastered across the beam using a step size of 25 µm and a dwell time of 300 ms per pixel. The incident X-ray energy was 13 keV, and fluorescence photons were collected with a single-element Vortex detector 45° from the sample (90° from the incident beam). Fluorescence signals were integrated using two different methods. First, regions of interest (ROIs) in the fluorescence spectrum were centered on the *K*α emission energy, and counts were accumulated in each bin at the beamline (As, Ca, Cl, Cu, Fe, K, Mn, P, S, Si and Zn). This method cannot separate overlapping fluorescent emission lines and thus could be subject to error, particularly for lighter elements where overlapping emission lines are more common. Second, fluorescence spectra were fit during data post-processing at each pixel using the *PyMCA* module within the *Microprobe Analysis ToolKit* [*SMAK* version 1.4 (Webb *et al.*, 2011 ▸; Solé *et al.*, 2007 ▸)]. Additional elements not previously included in the ROIs were identified and included (*e.g.* Ar present in the atmosphere). Fluorescence spectral fitting can also deconvolve overlapping elements and account for other potential artifacts, such as pile-up (Solé *et al.*, 2007 ▸). For XRF calibration, thin films with known elemental concentrations (Micromatter, Surrey, BC, Canada) were measured during each experiment while keeping all setup and detector parameters constant. Each film contained 20–75 µg cm^−2^ of a single compound vacuum deposited onto a 6 µm-thick mylar film.

After data collection, samples were post-processed using *SMAK*. Elevated concentrations of Cl in the ep­oxy allowed for the demarcation of the background from the grain. The fluorescence signal at each pixel was normalized by the incident beam intensity, and the average intensities of elements in the background were subtracted for each element. Normalized intensities from the standard thin films were used to quantify the sample intensities using a one-point calibration. Standard thin films and samples were kept at the same distance from the detector to account for signal attenuation in the air. Areal concentrations were converted to mass concentrations using an assumed density of 1.35 g cm^−3^ for rice grain and corrected for X-ray attenuation in the material assuming rice grain was similar in composition to ‘soft tissue’ (a preset material in *SMAK*). For comparison with ICP-MS data, elemental concentrations were averaged within the grain. For K and Cu, thin films were not analyzed during one of the experiments, so *n* = 29, whereas, for all other elements, *n* = 50.

To create quantitative maps for elements of interest, the quantitative SR-µXRF data were corrected by the regression line between SR-µXRF and ICP-MS data. In addition, Gaussian blurring was used to lightly smooth the image with a standard deviation of 0.8 in a neighborhood of 3 pixels. The image was also stretched in the *x* direction to correct for the sample being held at 45° relative to the incident beam and the detector.

#### 2.1.4. Statistical analysis

All regression analyses were performed using *SAS* 9.4 and *PROC REG*. Binary flags were included for grain orientation and synchrotron experiments. The experiment flag was never significant and was removed from the models, indicating that the elemental calibration could account for variability in beamline characteristics between experiments. Residuals did not show evidence of heteroscedasticity or non-normality.

SR-µXRF method detection limits (MDLs) were estimated following the approach of Twining *et al.* (2003 ▸). Fluorescence counts, a discrete random event, follow a Poisson distribution. For simplicity, the background area and sample area were considered equivalent,

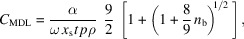

where *C*_MDL_ is the estimated MDL (mg kg^−1^), ω is the fluorescence yield as calculated from the standard (counts s^−1^)/(mg cm^−2^), *x*_s_ is the thickness of the sample (cm), *t* is the dwell time (s pixel^−1^), *p* is the number of pixels in the sample, ρ is the sample density (kg cm^−3^), α is a dimensionless attenuation factor to correct for sample thickness and *n*_b_ is the estimated number of counts from the background. Additional details of the derivation are provided in the supporting information.

## 3. Results

### 3.1. Agreement between SR-µXRF and ICP-MS

The agreement between average SR-µXRF and ICP-MS grain concentrations was minimally affected by grain orientation and fluorescence integration method (Table S1 of the supporting information). Linear models with ICP-MS grain concentration, grain orientation and their interaction found that grain orientation *p*-values remained >0.1 for all elements tested, and the interaction between grain orientation and ICP-MS grain concentration was insignificant for most elements (Table S1 of the supporting information). Thus, the optimal model for SR-µXRF was a simple linear regression with ICP-MS concentration as the descriptor. Comparing fluorescence integration methods, neither *p* values nor model fit statistics appreciably differed between methods for most elements, with only sulfur exhibiting differences in distribution (Fig. S1 of the supporting information) and moderate differences in fit slope (Table S1). Because minimal differences were observed between fluorescence integration methods, the simpler and less time-consuming ROI method was used throughout.

**Figure 1:**
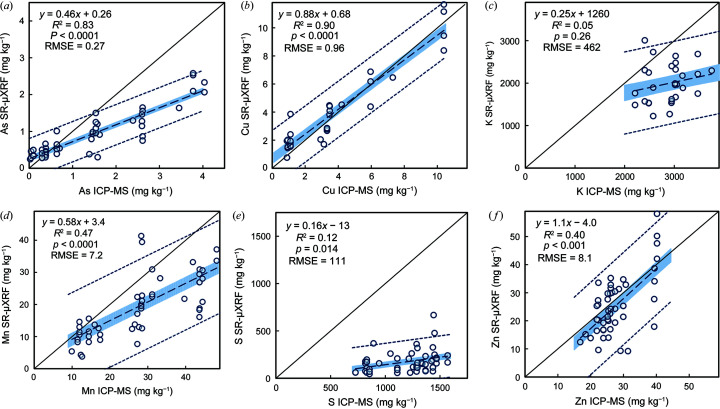
Simple linear regression fits between ICP-MS and SR-µXRF average elemental concentrations (*a*) As, (*b*) Cu, (*c*) K, (*d*) Mn, (*e*) S and (*f*) Zn in rice grains. The shaded region indicates the 95% confidence interval for the best-fit line. The dashed lines indicate the 95% prediction interval for the best-fit line. For K and Cu, *n* = 29; for all other elements, *n* = 50.

Rice grain concentrations measured by SR-µXRF and ICP-MS were more strongly in agreement for higher-*Z* elements than for lower-*Z* elements (Fig. 1 ▸). Copper and zinc exhibited the highest agreement between SR-µXRF and ICP-MS, with slopes not significantly different than unity. Arsenic and manganese also showed a significant correlation between SR-µXRF and ICP-MS and a high level of accuracy (As slope = 0.46, Mn slope = 0.58). Potassium and sulfur showed a weak correlation between SR-µXRF and ICP-MS data (*R* ^2^ < 0.1 or *p* > 0.1). Both iron and phospho­rous did not show a correlation between the methods (Fig. S2 of the supporting information). Calcium was observed in SR-µXRF measurements, but poor ICP-MS recovery (12%) prevented comparison. Silicon was also observed in SR-µXRF measurements, but its concentration was not certified in the reference material, and its presence in the quartz slide hindered quantification.

### 3.2. Reproducibility

**Figure 2:**
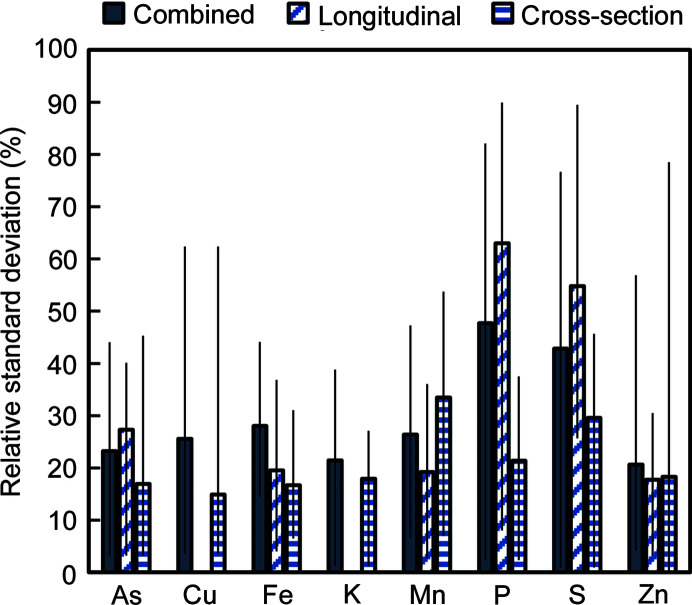
Reproducibility of SR-µXRF measurements of replicate sample scans for grains sectioned longitudinally, cross-sectionally or combining the two sectioning types. The combined values include replicates across and within section types. Note that replicate scans have biological variability as each scan is from a different grain from an individual plant. Error bars denote the maximum and minimum values measured (*n* = 12 for combined, *n* = 8 for longitudinal and cross-section). Missing bars indicate that no data were available.

The use of multiple grains placed in a single thin section allowed for method reproducibility to be tested on a subset of samples. Of the 33 unique treatment combinations, multiple grains (2–4) were analyzed for 12 treatment combinations. For each, the relative standard deviation (RSD) was used to measure reproducibility. Note that this also includes any variability between grains from an individual plant, making these biological replicates rather than analytical replicates. The overall average RSD for the elements of interest was 29%. Most elements were more reproducible than this, apart from P and S (Fig. 2 ▸). Separating by section type (longitudinal versus cross-section) revealed minimal differences in reproducibility except for P and S, in which cross-sectioned grains showed improved reproducibility compared with longitudinally sectioned grains. The maximum RSD never exceeded 100% for these biological replicates and stayed within 50% for As, Fe, K and Mn (combined data).

### 3.3. Method detection limits

Estimated MDLs were generally less than 1 mg kg^−1^ and were lower for high-*Z *elements (Table S2). Increasing the number of pixels in the scan slightly decreased the MDLs. Dwell time was held constant to avoid affecting the MDL. Except for As, all average grain concentrations were more than one order of magnitude above their corresponding MDL. For As, the median sample was one order of magnitude greater than the MDL, and seven samples were less than the MDL.

### 3.4. Quantitative SR-µXRF maps

**Figure 3:**
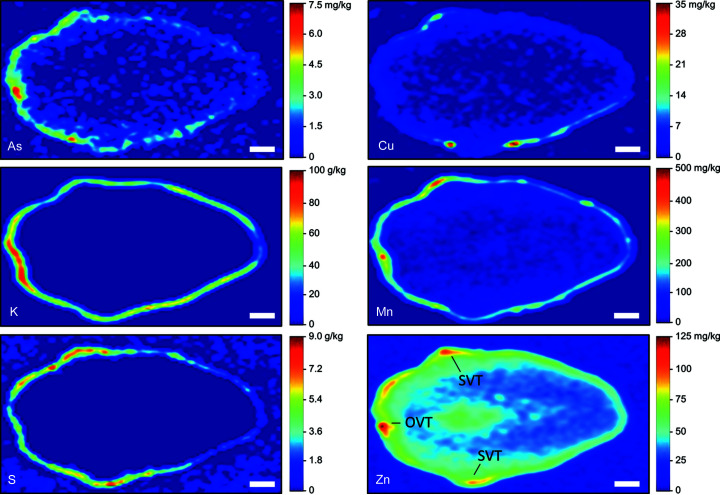
Quantitative SR-µXRF elemental concentrations in the cross-section of a rice grain grown in soil with low levels of As (∼5 mg kg^−1^). Scale bar denotes 300 µm. OVT: ovular vascular trace; SVT: stylar vascular trace.

**Figure 4:**
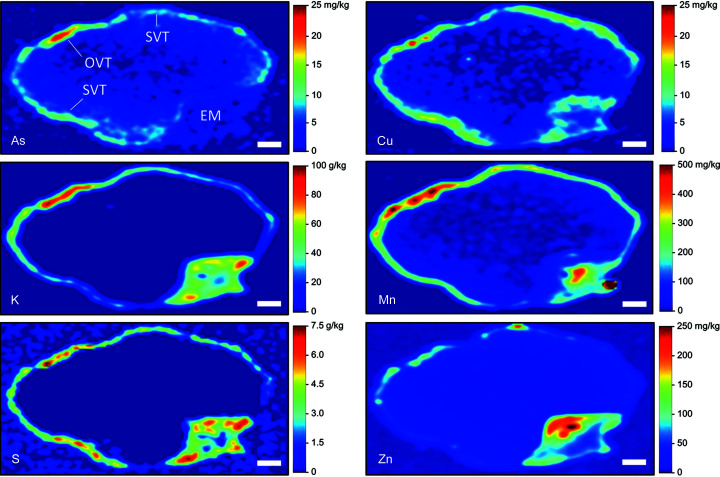
Quantitative SR-µXRF elemental concentrations in the cross-section of a rice grain grown in soil with elevated levels of As (∼24 mg kg^−1^). Scale bar denotes 300 µm. EM: embryo.

Using the calibrated SR-µXRF data (*i.e.* Fig. 1 ▸), quantitative concentration maps were generated for selected grains. Fig. 3 ▸ shows a cross-section of a grain obtained from a field site with low levels of soil As [∼5 mg kg^−1^ (Limmer, Mann *et al.*, 2018 ▸)]. Several elements were localized in the ovular vascular trace (OVT), stylar vascular trace (SVT) and/or the bran layer. Arsenic, manganese and zinc were highly concentrated in the OVT, with concentrations of ∼7, 500 and 100 mg kg^−1^, respectively. In this grain, only Zn, and to some extent As, showed substantial accumulation in the endosperm. A cross-section of another grain from the same treatment, but with the section taken through the embryo, showed a similar accumulation of most elements in the bran (Fig. S3). However, the OVT and SVT were not apparent, and K, Mn, S and Zn accumulated in the embryo. Interestingly, concentrations of elements in the embryo (Fig. S3) were of the same order of magnitude as concentrations in the OVT in Fig. 3 ▸. In contrast to these grains grown under low-background As, Fig. 4 ▸ shows a cross-section from a grain exposed to high soil As (∼24 mg kg^−1^) in a pot study (Teasley *et al.*, 2017 ▸). The deformed grain had localized concentrations of elements similar to those from the low-As treatment, except for As. Arsenic in the OVT was ∼20 mg kg^−1^, approximately 3× the concentration of As in the OVT of the low-As grain (Fig. 3 ▸).

**Figure 5:**
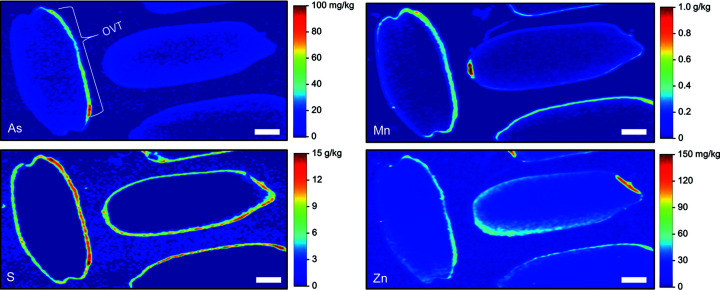
Quantitative SR-µXRF elemental concentrations in the longitudinal section of rice grains grown in soil with elevated levels of As (∼24 mg kg^−1^). Scale bar denotes 1 mm.

Concentration maps for longitudinally sectioned grains show variable concentrations of elements depending on whether the section was included the OVT. For example, Figs. 5 ▸ and S4 show one grain where the OVT was included in the section, resulting in high concentrations of As, Mn and Zn relative to other grains where the OVT was not evident. Note that As was not uniformly concentrated throughout the OVT, with a hot spot approaching 100 mg kg^−1^ As.

## 4. Discussion

### 4.1. Agreement between SR-µXRF and ICP-MS

**Figure 6:**
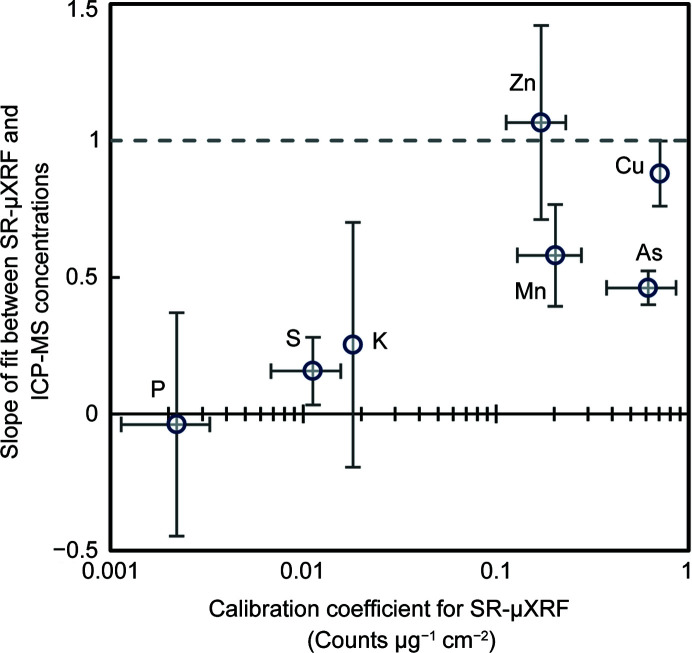
Comparison of SR-µXRF accuracy against the sensitivity of SR-µXRF for selected elements, showing that SR-µXRF is low for elements when SR-µXRF sensitivity is low. The dashed line is the ideal slope of unity, indicating 1:1 agreement between SR-µXRF and ICP-MS concentrations. Error bars in the *x* direction denote the range (*n* = 2) in SR-µXRF sensitivity between synchrotron experiments when available. For the *y* axis, the error bars are the 95% confidence intervals for the slope.

At this hard X-ray beamline, the agreement between SR-µXRF and ICP-MS concentrations was better for high-*Z* elements than low-*Z* elements. Fig. 6 ▸ shows the relationship between the SR-µXRF calibration coefficient (*i.e.* the fluorescence yield) and the slope of the SR-µXRF/ICP-MS fit line. Elements with larger calibration coefficients had slopes close to the ideal value of unity (*i.e.* Mn, Cu and Zn). Arsenic was a notable exception, likely arising from the concentrations being close to the SR-µXRF MDL. Iron (not shown) would be expected to perform well, with a calibration coefficient of ∼0.25 counts µg^−1^ cm^−2^. However, SR-µXRF drastically overestimated the concentration of Fe in the grain (Fig. S2), an artifact we believe resulted from Fe contamination during the sectioning process. Lighter elements, such as S and K, had small SR-µXRF calibration coefficients and slopes much below unity. Phospho­rous performed particularly poorly (Fig. S2) with a slope near zero (Fig. 6 ▸), suggesting either P was not detectable or that the 3D distribution of P could not be captured with 2D sections. Most elements had intercepts with confidence intervals that included zero, indicating adequate agreement at low concentrations, with the accuracy limited by the SR-µXRF MDL. For elements with slopes near unity and intercepts near zero, quantitative SR-µXRF values could be used directly without adjustment. For other elements, calibration coefficients were necessary to generate accurate quantitative maps.

The poor performance of SR-µXRF for S and P likely arose from several factors. First, the beamline operating at 13 keV was not ideal for low-*Z* elements, as shown by the small calibration coefficients. With increasing energy far above the edge, the X-ray absorption cross-section decreases, decreasing the fluorescence yield. Low-*Z* elements are also intrinsically less efficient producers of fluorescent X-rays due to their higher yield of auger electrons, further decreasing fluorescence yield (Hubbell *et al.*, 1994 ▸). Additionally, the sample matrix and air strongly attenuate the fluorescent X-rays emitted from these elements. As assumed here, the matrix for fluorescence attenuation correction may be incorrect or not homogeneous. SR-µXRF is typically explicitly used to investigate spatial variability in elemental composition, so assumptions of compositional uniformity can be problematic. This is most problematic when the fluorescence energy of one element is close to (and above) the excitation edge of other elements and when these elements are co-localized at high concentrations (Fendorf & Sparks, 1996 ▸). This may explain the poor performance of K (due to S and Cl), S (due to Si and P) and P (due to Si and Mg) and highlights the need for fluorescence attenuation corrections that vary in space and/or adaptively adjust with measured elemental intensities. Finally, errors can arise from 3D heterogeneities not captured in 2D thin sections, as some of these elements are present in the embryo at substantially high concentrations. Thus, using quantitative SR-µXRF is likely to perform better for high-*Z* elements with higher fluorescence yields, lower attenuation by air, fewer strongly absorbing elements (*e.g.* within 1 keV) and at beamlines designed for the element(s) of interest. Sample-specific factors, such as the 3D heterogeneity and the colocalization of similar elements, will also strongly affect the success of quantitative SR-µXRF.

### 4.2. Elemental distribution in rice grains

The false-color images presented here agree with the literature that many elements primarily accumulate in the bran layer of rice, including Ca, Cu, K, Fe, Mn and P (Kyriacou *et al.*, 2014 ▸; Lombi *et al.*, 2009 ▸; Lu *et al.*, 2013 ▸; Meharg *et al.*, 2008 ▸; Sakai *et al.*, 2015 ▸). Others have also shown Fe, Mn and inorganic As accumulation in the OVT (Carey *et al.*, 2011 ▸). Here we found that As, Mn and Zn most strongly accumulated in the OVT. Previous work has shown that both S and Zn are concentrated in the bran layer but can also slightly penetrate the endosperm to varying degrees (Kyriacou *et al.*, 2014 ▸; Lombi *et al.*, 2009 ▸; Meharg *et al.*, 2008 ▸; Sakai *et al.*, 2015 ▸). In this work, we observed grains where S was strongly localized to the bran (*e.g.* Fig. 4 ▸) and grains where S penetrated the endosperm ( *e.g.* Fig. S4), suggesting the extent of S localization may vary. Of the elements we quantified, Zn was most able to penetrate the endosperm, although much of the Zn remained in the bran. Additionally, minor longitudinal heterogeneity appears in Zn concentrations in the endosperm, with more Zn penetrating the endosperm near the awn (Fig. 5 ▸). As previously reported, Cd and Ni are homogeneous throughout the grain, although there are limited observations of these elements due to low concentrations in the grain and the high energy needed to excite the *K*-edge for Cd (Meharg *et al.*, 2008 ▸); thus, Cd may be better suited for LA-ICP-MS mapping. Lu *et al.* (2013 ▸) found Ca, Fe, K, Mn and Zn accumulated in the embryo, with Fe and Ca mainly in the scutellum and Zn mainly in the plumule and radicle. We observed Cu, K, Mn, S and Zn in the embryo but not As. Importantly, the distribution of elements can also be affected by elemental speciation. There are several reports of such effects for As and Se, with the organic forms dispersed throughout the grain while the inorganic forms accumulate in the bran and OVT (Carey *et al.*, 2012 ▸; Zheng *et al.*, 2011 ▸; Zheng, Li *et al.*, 2013 ▸; Limmer & Seyfferth, 2022 ▸). Quantitative SR-µXRF can theoretically separate species when species can be identified by differences in their XANES spectra, but we are unaware of any such reports that quantitatively resolve these species.

Of all the rice plant parts, elemental concentrations and distribution in grain most directly affect human health. Grain concentration and speciation of toxic elements, such as arsenic, have health-based limits due to exposure risks (Meharg *et al.*, 2009 ▸). Conversely, Fe and Zn deficiencies affect billions of people globally, resulting in efforts to increase grain concentrations of these essential elements (Slamet-Loedin *et al.*, 2015 ▸). In both cases, human exposure depends on the extent of elemental localization in the bran and the extent of polishing. For Fe and Zn, chelation with P in the form of phytic acid strongly reduces bioavailability to consumers (Perera *et al.*, 2018 ▸). Thus, quantitative elemental maps could provide colocalization information and molar ratios of metal:phytic acid. Additionally, because trace elements are a minor fraction of osmolytes in the phloem transported to filling grains, the grain concentration of such elements is governed by the phloem concentration of each element (Zhang *et al.*, 2007 ▸). Collectively, methods to quantify the localization of elements in grain could inform studies of the regulation of elemental homeostasis in various plant tissues and aid in protecting human health.

### 4.3. Application considerations

The application of quantitative SR-µXRF involves several considerations, many of which are also of interest for qualitative SR-µXRF and SR-nanoXRF [see Donner *et al.* (2013 ▸) for a comprehensive discussion of qualitative SR-µXRF considerations]. Fundamental facility considerations include the suitability of the beamline, particularly factors such as the incident beam energy, the size of the incident beam relative to the size of the features of interest and the available detector(s). Elements of interest must have edges (ideally *K*-edges for most elements) below the incident beam energy available, although sensitivity will decrease as the energy increases farther from the edge. The size of the incident beam must be smaller than the size of the features of interest but large enough to map the desired area in a reasonable amount of time. Finally, the detector must be sensitive enough at the desired energy to measure the low concentrations of the desired element. An He sample chamber is likely to be necessary for low-*Z* elements to minimize fluorescent X-ray attenuation. Method detection limits, while valuable, are a function of the aforementioned factors and factors discussed below, limiting their direct application to other situations. Regardless, the MDLs in Table S2 and the literature (*e.g.* Mihucz *et al.*, 2010 ▸) can provide a first-order approximation in other settings. Performing quantitative SR-XRF at finer spatial resolution (*e.g.* SR-nanoXRF) is likely to become increasingly difficult as the volume probed by the X-ray becomes more heterogenous at the nano-scale. This is perhaps best addressed by making very thin sections [*e.g.* 2 µm (de Samber *et al.*, 2020 ▸)] to minimize the attenuation of fluorescent X-rays by the matrix.

Sample preparation is also an important consideration. Because heterogeneous samples are of most interest in SR-µXRF and 2D SR-µXRF sections are most frequently analyzed, determining how to reduce a 3D sample to a 2D sample is crucial. Ideally, the sample should be homogeneous across the third dimension, allowing a 2D thin section to be made. If 3D information is needed, microtomography or confocal SR-µXRF may be better alternatives. Rice grains, except for the embryo, are largely homogeneous along the length of the grain, making cross-sections ideal 2D thin sections. Thus, grain cross-sections can map elemental changes from the bran into the endosperm but cannot provide elemental information along the length of the grain. Furthermore, cross-sections are generally symmetric across the OVT, thereby potentially further minimizing the amount of sample to scan. Determining the optimum sample thickness depends on several factors. Although thicker samples increase fluorescence for high-*Z* elements, minimal improvement occurs for low-*Z* elements. For example, in a 30 µm-thick rice grain section, the S fluorescence is only representative of a 10 µm-thick sample due to fluorescence attenuation within the sample. Additionally, high-*Z* elements in exceptionally thick samples, such as a whole rice grain (*e.g.* Zheng *et al.*, 2011 ▸), will complicate interpretation as the 3D sample is projected onto a 2D plane, combining signals from the bran and the endosperm. Even sections of moderate thickness (*i.e.* 1 mm) can result in blurred images due to heterogeneity in depth (Carey *et al.*, 2010 ▸). Thick samples also complicate the comparison of low-*Z* and high-*Z* elements when samples are not homogeneous with depth, as the fluorescence signal is practically a surface measurement for low-*Z* elements and a depth-integrated measurement for high-*Z* elements.

An additional consideration is the number of replicate samples to analyze. Because beam time is limited, analyzing replicate samples is unfortunately not often a priority. Thus, conclusions about an entire population may be based on a single rice grain. Analysis of replicate grains here showed that the RSD of mean grain elemental concentrations averaged ∼30% but was considerably higher for P and S, which are low-*Z* elements that are better investigated using a different beamline and/or different experimental parameters (*e.g.* He atmosphere). However, this RSD does not consider changes in the localization of elements, only the average concentration in a grain, and thus may be a liberal estimate of the variation between replicate samples. Care should also be taken in selecting samples to examine in detail. For example, if several grains are scanned coarsely for a trace element, and the grain with the highest fluorescence is studied at a finer resolution (*i.e.* ‘hot spot’ selection), this grain is likely not representative of the population.

Given the results of this work, the application of quantitative SR-µXRF to other plant parts seems possible. However, more care must be taken when working with hydrated samples by either collecting data very quickly (*e.g.* with a Maia detector) or under cryogenic conditions (Castillo-Michel *et al.*, 2017 ▸) to avoid sample distortion during dehydration. Additionally, plant parts must be large enough to enable the measurement of bulk concentrations for validation. In rice, the nodes are an area of interest because of the heterogeneity and the high concentrations of metals sequestered (Yamaji & Ma, 2014 ▸). Qualitative SR-µXRF has already been performed in the nodes (Chen *et al.*, 2015 ▸; Moore *et al.*, 2014 ▸; Yamaguchi *et al.*, 2012 ▸; Zheng *et al.*, 2011 ▸), although quantitative SR-µXRF may be complicated by the 3D heterogeneity of the nodes. Nevertheless, quantitative SR-µXRF would be a powerful tool in the nodes, allowing measurement of sequestered metal(loid) and sulfur (*e.g.* phytochelatins) concentrations in this critical plant organ. Using quantitative SR-µXRF in other plant organs and plant parts will require validation through comparison with other accurate, quantitative techniques.

## Supplementary Material

Calibration statistics, derivation of method detection limit, comparison of fluorescence integration methods, additional elemental correlations and additional quantitative elemental maps. DOI: 10.1107/S1600577523000747/ok5087sup1.pdf
