# Supplementary material for: Evaluation of quantitative synchrotron radiation micro-X-ray fluorescence in rice grain
Source: J Synchrotron Radiat. 2023 Feb 15;30(Pt 2):407–16. doi: 10.1107/S1600577523000747 (PMC10000813; doi:10.1107/S1600577523000747)
Supplement: Supplementary file 1 [file s-30-00407-sup1.pdf]

JOURNAL OF SYNCHROTRON RADIATION

Volume 30 (2023)

Supporting information for article:

Evaluation of Quantitative Synchrotron Radiation micro-X-Ray Fluorescence in Rice Grain

Matt A. Limmer, Samuel M. Webb and Angelia L. Seyfferth

S1. Model Fit Statistics

Table S1 Model fit statistics for grain elemental concentrations measured by SR-µXRF with ICP-MS and grain orientation as descriptors. A significant grain orientation effect was not generally observed, so simple linear regression using ICP-MS elemental concentrations was selected as the optimal model. Fit statistics, including parameter estimate 95% confidence intervals (CI), are shown to the right. Minimal differences between fluorescence integration methods (Fluor. Int.) for all elements were observed. For Cu and K, n=29; for other elements, n=50.

Element	Fluor. Int.	p-values			Model statistics for simple linear regression with ICP-MS					
		ICP-MS	Grain Orient	ICP-MS * Grain Orient	RMSE	R^2	Slope Estimate	Slope CI	Intercept Estimate	Intercept CI
As	ROI	<0.0001	0.45	0.85	0.27	0.83	0.46	0.06	0.26	0.11
	PyMCA	<0.0001	0.35	0.53	0.19	0.86	0.37	0.04	0.18	0.08
Cu	ROI	<0.0001	0.13	0.0067	0.96	0.90	0.88	0.12	0.68	0.57
	PyMCA	<0.0001	0.16	0.15	1.3	0.76	0.70	0.16	-0.06	0.79
K	ROI	0.15	0.43	0.73	462	0.05	0.25	0.45	1260	1298
	PyMCA	0.39	0.29	0.53	548	0.006	0.10	0.53	1899	1542
Mn	ROI	<0.0001	0.28	0.94	7.2	0.47	0.58	0.19	3.4	5.0
	PyMCA	<0.0001	0.41	0.92	7.2	0.54	0.62	0.18	1.3	4.7
P	ROI	0.77	0.39	0.64	555	0.001	-0.038	0.41	1094	1327
	PyMCA	0.99	0.47	0.67	565	0.003	-0.081	0.41	1451	1353
S	ROI	0.0063	0.14	0.25	111	0.12	0.16	0.12	-13	152
	PyMCA	0.0001	0.55	0.14	260	0.22	0.54	0.29	-170	355
Zn	ROI	<0.0001	0.38	0.076	8.1	0.40	1.07	0.36	-4.0	11.3
	PyMCA	<0.0001	0.81	0.23	9.0	0.31	0.95	0.42	-2.5	11.7

S2. Method Detection Limit

We follow the approach of Twining et al. (2003), developing the MDL from Poisson statistics. For each element, the coefficient of variation (*CV*) is:

$$CV = \frac{(n_s + 2n_b)^{0.5}}{n_s} \tag{1}$$

Where n_s is the estimated number of counts from the sample and n_b is the estimated number of counts from a background area of equivalent size to the sample and counted over the same period.

The number of sample counts can be calculated as follows:

$$n_s = \frac{C_s \omega x_s t p \rho}{\alpha} \tag{2}$$

where C_s is the elemental concentration (mg/kg), ω is the fluorescence yield as calculated from the standard ((counts/s)/(mg/cm^2)), x_s is the thickness of the sample (cm), t is the counting time (s/pixel), p is the number of pixels in the sample, ρ is the sample density (kg/cm^3), and α is a dimensionless attenuation factor to correct for sample thickness.

Substituting equation 2 into equation 1 gives:

$$CV = \frac{\left(\frac{C_s \omega x_s t p \rho}{\alpha} + 2n_b\right)^{0.5}}{\frac{C_s \omega x_s t p \rho}{\alpha}} \tag{3}$$

$$\left(CV \frac{C_s \omega x_s t p \rho}{\alpha}\right)^2 = \frac{C_s \omega x_s t p \rho}{\alpha} + 2n_b \tag{4}$$

$$0 = C_s^2 \left(CV \frac{\omega x_s t p \rho}{\alpha}\right)^2 - \frac{C_s \omega x_s t p \rho}{\alpha} - 2n_b \tag{5}$$

$$C_s = \frac{\frac{\omega x_s t p \rho}{\alpha} + \left(\left(\frac{\omega x_s t p \rho}{\alpha}\right)^2 + 4\left(CV \frac{\omega x_s t p \rho}{\alpha}\right)^2 2n_b\right)^{0.5}}{2\left(CV \frac{\omega x_s t p \rho}{\alpha}\right)^2} = \frac{1 + (1 + 8CV^2 n_b)^{0.5}}{2CV^2 \frac{\omega x_s t p \rho}{\alpha}} \tag{6}$$

For $\mu = n_s = 3\sigma$, equivalent to a CV of 1/3, the MDL is:

$$C_{MDL} = \frac{1 + \left(1 + \frac{8}{9} n_b\right)^{0.5}}{\frac{2}{9} \cdot \frac{\omega x_s t p \rho}{\alpha}} = \frac{\alpha}{\omega x_s t p \rho} \frac{9}{2} \left[1 + \left(1 + \frac{8}{9} n_b\right)^{0.5}\right] \tag{7}$$

Table S2 Estimated method detection limits for SR-µXRF (mg/kg)

	Average	Maximum
**As**	0.056	0.14
**Cu**	0.034	0.066
**K**	1.3	2.6
**Mn**	0.10	0.23
**S**	0.80	1.6
**Zn**	0.059	0.27

S3. Comparison of Elemental Concentration Maps by Fluorescence Integration Method

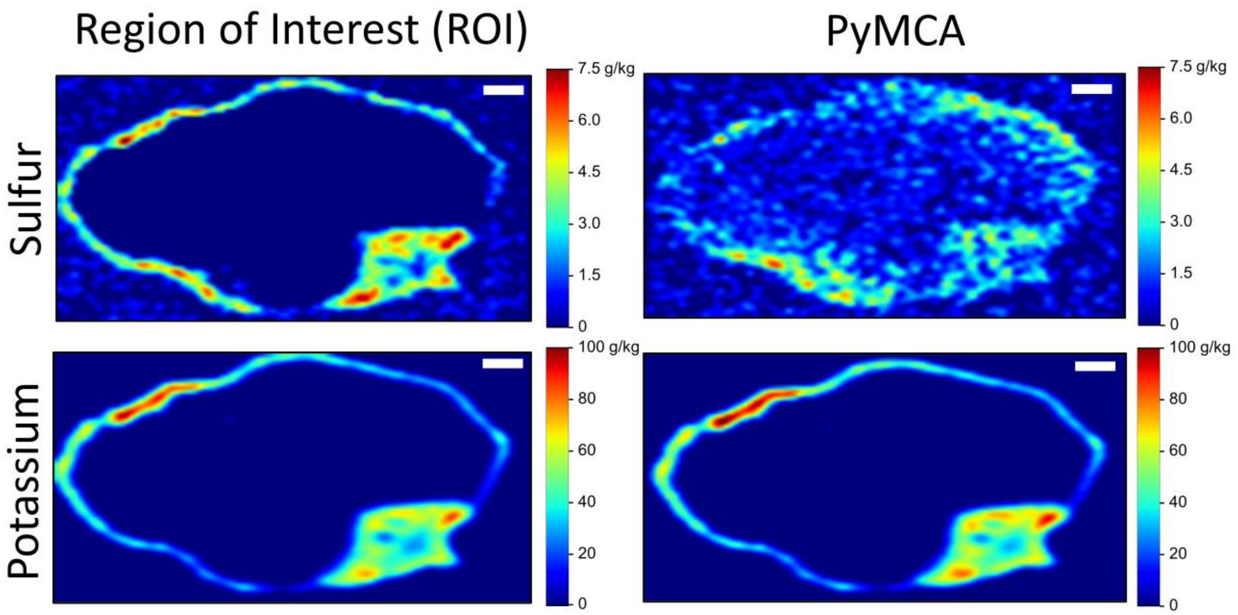

Figure S1 SR-µXRF elemental concentration maps for sulfur and potassium of the rice grain shown in Fig. 4 after integrating fluorescence counts using ROI or PyMCA. Overall concentrations are similar, but integration by PyMCA shows more penetration of S into the grain, although data appear to be approaching the detection limit. In contrast, K shows no appreciable difference between integration methods. Scale bar is 300µm.

S4. Additional Correlations

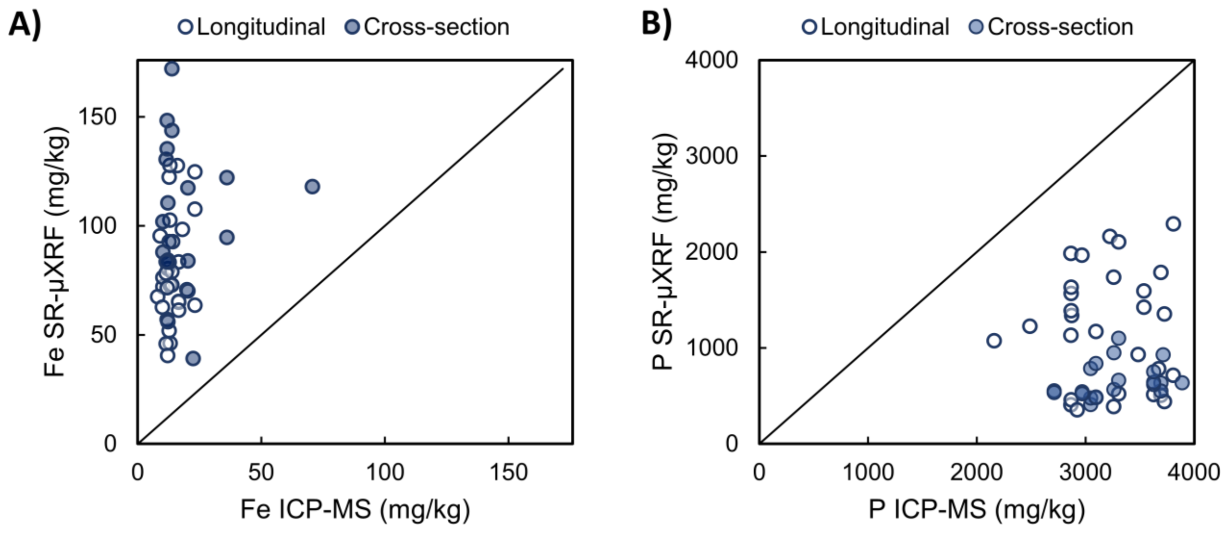

Figure S2 Correlation between ICP-MS and SR-µXRF average elemental concentrations for A) Fe and B) P in rice grains. No significant correlation was observed between XRF and ICP-MS concentrations.

S5. Additional Maps

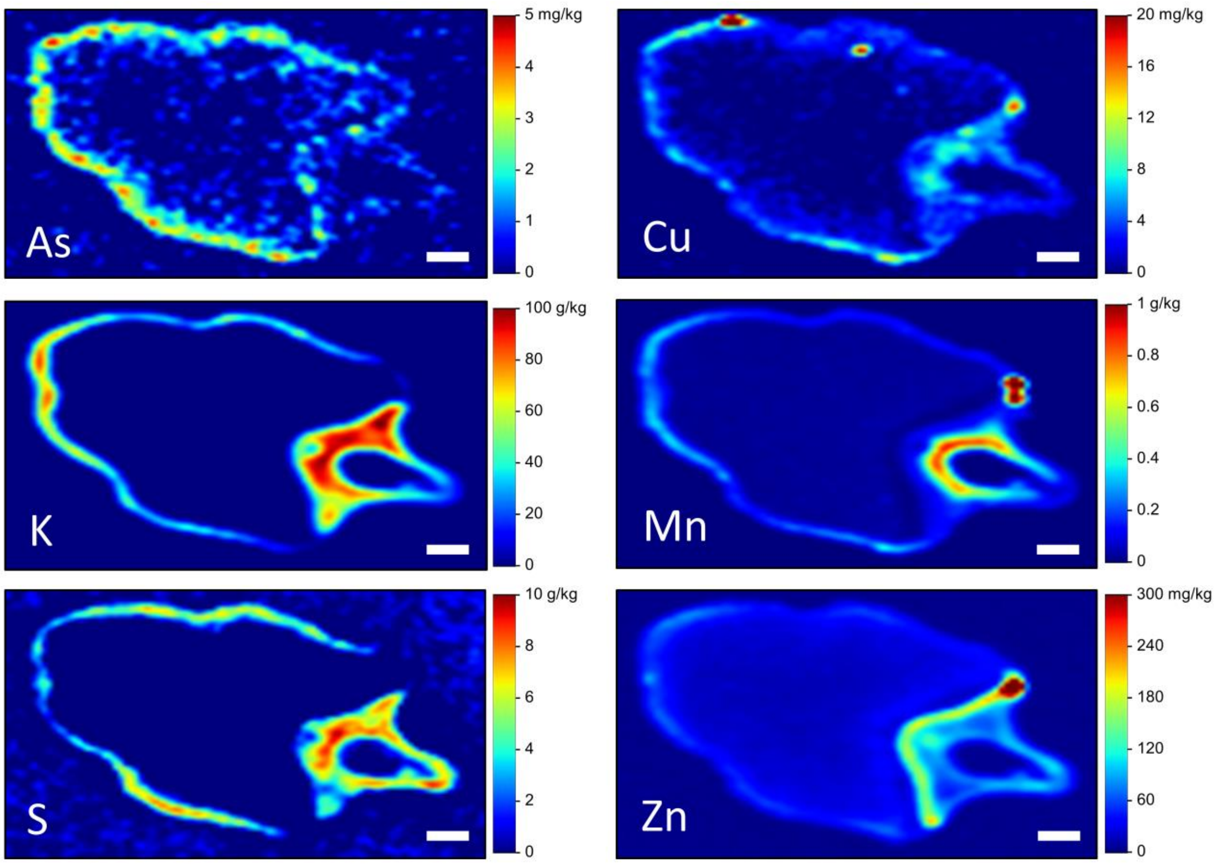

Figure S3 Quantitative SR-µXRF elemental concentrations in a cross-section of a soil-grown rice grain. Note that this grain is from the same treatment as the grain in Figure 3. Scale bar denotes 300 µm.

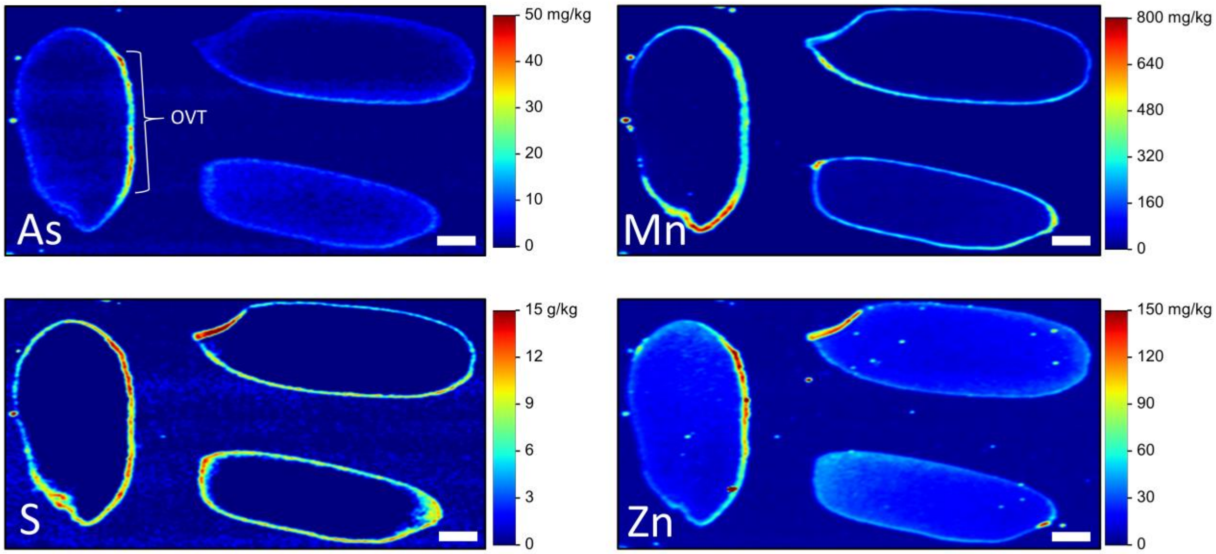

Figure S4 Quantitative SR-µXRF elemental concentrations in the longitudinal-section of rice grains grown in soil with elevated levels of As (~24 mg/kg). Scale bar denotes 1 mm. OVT: ovular vascular trace.
